# Pronto: A Multi-Sensor State Estimator for Legged Robots in Real-World Scenarios

**DOI:** 10.3389/frobt.2020.00068

**Published:** 2020-06-05

**Authors:** Marco Camurri, Milad Ramezani, Simona Nobili, Maurice Fallon

**Affiliations:** ^1^Dynamic Robot Systems, Department of Engineering Science, Oxford Robotics Institute, University of Oxford, Oxford, United Kingdom; ^2^School of Informatics, Institute of Perception, Action and Behavior, University of Edinburgh, Edinburgh, United Kingdom

**Keywords:** legged robots, state estimation, sensor fusion, visual odometry, iterative closest point (ICP), extended kalman filter (EKF)

## Abstract

In this paper, we present a modular and flexible state estimation framework for legged robots operating in real-world scenarios, where environmental conditions, such as occlusions, low light, rough terrain, and dynamic obstacles can severely impair estimation performance. At the core of the proposed estimation system, called Pronto, is an Extended Kalman Filter (EKF) that fuses IMU and Leg Odometry sensing for pose and velocity estimation. We also show how Pronto can integrate pose corrections from visual and LIDAR and odometry to correct pose drift in a loosely coupled manner. This allows it to have a real-time proprioceptive estimation thread running at high frequency (250–1,000 Hz) for use in the control loop while taking advantage of occasional (and often delayed) low frequency (1–15 Hz) updates from exteroceptive sources, such as cameras and LIDARs. To demonstrate the robustness and versatility of the approach, we have tested it on a variety of legged platforms, including two humanoid robots (the Boston Dynamics Atlas and NASA Valkyrie) and two dynamic quadruped robots (IIT HyQ and ANYbotics ANYmal) for more than 2 h of total runtime and 1.37 km of distance traveled. The tests were conducted in a number of different field scenarios under the conditions described above. The algorithms presented in this paper are made available to the research community as open-source ROS packages.

## 1. Introduction

Legged robotics is rapidly transitioning from research laboratories into the real world, as demonstrated by the recent introduction of several commercial quadruped platforms.

To be truly useful, legged robots must be able to reliably and rapidly navigate across rough terrain and be stable in the presence of disturbances, such as slips or pushes. They must also be able to perceive and manipulate the environment whilst avoiding collisions with obstacles and people.

None of these tasks can be accomplished without the ability to accurately and robustly estimate the pose and velocity of the robot (i.e., its state) in real time using only onboard sensors and computers. The robot's state is used to plan and track body trajectories, to balance and recover from external disturbances, and to map the environment and navigate through it.

To achieve a satisfactory level of accuracy, proprioceptive and exteroceptive sensor fusion is necessary, giving rise to the problem of synchronization and latency between the different signals coming from each sensor.

Migrating from the controlled environment of a laboratory to the real operating conditions of industrial applications (e.g., oil rig platform inspection or mine exploration) makes the task even more challenging, as it requires extra effort to robustify the estimation algorithm against unknown situations and long periods of continuous operation without human intervention.

In this paper, we demonstrate how inertial, kinematic, stereo vision, and LIDAR sensing can be combined to produce a low-latency and high-frequency state estimate that can be directly used to control state-of-the-art humanoids and dynamic quadrupeds. In turn, this estimate can be used to build accurate maps of the robot's immediate environment and to enable navigational autonomy and manipulation.

**Figure 1:**
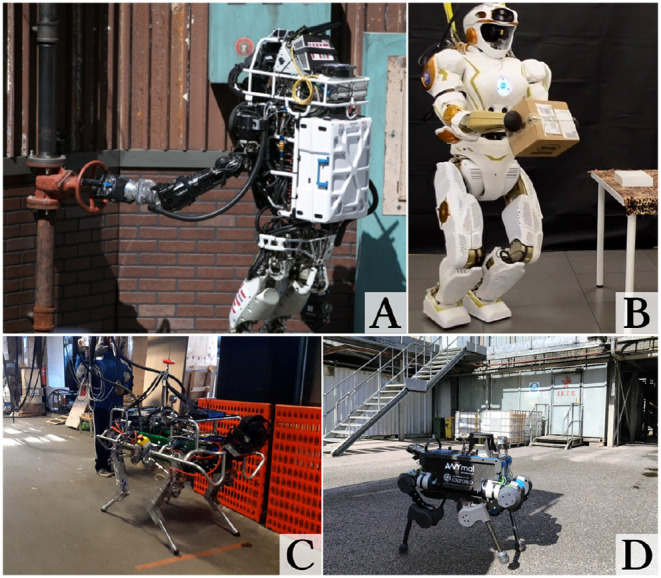
The Atlas **(A)** and Valkyrie **(B)** humanoid robots and the HyQ **(C)** and ANYmal **(D)** dynamic quadruped robots. Sources: MIT, University of Edinburgh, Istituto Italiano di Tecnologia (IIT), University of Oxford.

This contribution is the first such research to provide an open source implementation of a fully integrated state estimation system performing sensor fusion of IMU, kinematics, stereo vision, and LIDAR on four different legged platforms: the NASA Valkyrie and Boston Dynamics Atlas humanoids, and the IIT HyQ and ANYbotics ANYmal quadrupeds (Figure 1). Another key achievement in comparison with the state of the art is demonstrating that the algorithm can be used to close the loop with the controller.

### 1.1. Contribution

This paper combines previous works focused on the individual platforms including state estimation on Atlas in Fallon et al. (2014), an extension to the HyQ quadruped and incorporation of vision in Nobili et al. (2017a), and further evaluation of LIDAR localization in Nobili et al. (2017b).

The paper provides a complete and coherent overview of the method with (a) a comprehensive and updated survey of state estimation methods for legged robots; (b) additional experimental results on the ANYmal quadruped platform; (c) a more detailed description of the overall estimation method and architecture.

Furthermore, we release the Pronto state estimator, FOVIS Visual Odometry, and AICP LIDAR odometry modules as open-source ROS packages for the research community.

## 2. Related Work

The literature on state estimation for legged robots can be classified according to several criteria: the type of sensors used (proprioceptive, exteroceptive, or both); the output frequency (at control rate, e.g., 400 Hz or camera/LIDAR rate, e.g., 10 Hz); state definition (pose, velocity, joint states, contact points, etc.); the presence of loop closures (odometry vs. SLAM); the degree of marginalization of past states (from filtering to full batch optimization). Finally, if there is fusion of proprioceptive and exteroceptive signals, this can be performed in a *loosely* or *tightly* coupled manner.

In this section, we divide the related work into three main categories: *proprioceptive state estimation*, which includes filtering methods to fuse only the high-frequency signals, such as IMU and kinematics; *multi-sensor filtering*, which covers filtering methods with proprioceptive and exteroceptive sensor fusion; *multi-sensor smoothing*, which typically involve fusion of Visual Odometry (VO), IMU, and kinematics in a tightly coupled manner within probabilistic graphical model frameworks, such as factor graphs.

### 2.1. Proprioceptive State Estimation

Nearly all modern legged robots are equipped with IMUs, encoders, and force/torque sensors. Since these devices provide low-dimensional signals at high frequencies (250–1,000 Hz), they are the first to be fused for a smooth (although drifting) state estimate, useful for control purposes. Since real-time safety is paramount for controllers, most methods are based on the Kalman filter (section 2.1.1) or lightweight optimization (section 2.1.2).

#### 2.1.1. Kalman Filtering

Bloesch et al. (2012) were the first to propose an EKF-based state estimator method that did not depend on a specific type of gait or number of legs. The filter used IMU signals (linear acceleration and angular velocity) as inputs to be integrated for the process model. The state included the pose and velocity of the robot, as well as IMU biases and foot contact locations. In this way, they could define leg odometry measurements from the forward kinematics of the feet in stable contact with the ground. Their work was implemented on the StarlETH robot and tested in short indoor experiments. Shortly thereafter, Rotella et al. (2014) adapted the same method to humanoid platforms by including the ankle joint and the foot orientations in the state vector.

An important aspect of humanoid robot state estimation is the distance between the Center of Mass (CoM) and the feet, which is larger than for quadruped platforms. In humanoids, the flexibility of the links is therefore not negligible and can lead to falls when the CoM is incorrectly estimated to be inside the relatively small support polygon given by the robot's footprints. Xinjilefu et al. (2015) explicitly estimated the CoM offset using an inverted pendulum model to infer modeling error and/or unexpected external forces. In contrast, the approach of Koolen et al. (2016) modeled the elasticity of their robot's leg joints to better distribute error.

The above methods integrated the kinematics as position constraints. An alternative approach is to use differential kinematics in addition to forward kinematics to create linear velocity measurements, which are then integrated within the filter to get consistent position estimates. Bloesch et al. (2013) applied this approach again on the StarlETH quadruped robot. Since angular velocity from the IMU appeared on both the inertial process model and the measurement update, the authors proposed the use of the Unscented Kalman Filter (UKF) instead of an EKF to better handle the correlation between the joint and gyroscope noises.

Fallon et al. (2014) used the same elasticity model as Koolen et al. (2016) and integrated leg odometry as velocity measurements on the Atlas robot. Since the EKF models the measurements as Gaussian, non-linearities, such as slippages or impacts are not captured by the filter noise model. Therefore, special care was taken to ignore invalid contacts by classifying the outputs from the contact sensors in the feet.

When foot sensors are unavailable, the contact feet are detected by thresholding the Ground Reaction Forces (GRF), which are estimated from the joint torques. Camurri et al. (2017) proposed a method that evaluates GRF discontinuities to discard invalid leg odometry velocity measurements on the HyQ quadruped robot. To better detect the feet in contact, they also proposed a logistic regression method to learn the optimal GRF threshold on different gaits. A different approach, based on Hidden Markov Model (HMM), was adopted by Jenelten et al. (2019) for slip recovery on the ANYmal robot. In this case, the probability of contact for each leg was determined from dynamics and differential kinematics.

#### 2.1.2. Optimization

Kalman-based filtering has been preferred over more sophisticated methods because of its simplicity and low computational expense. However, recent technological progress has made optimization-based methods feasible to use. These methods can overcome some limitations, such as the need to define a process model even when unfit for the application. Indeed, the widely adopted EKF inertial process model approximates the robot to a ballistic missile, while optimization methods could incorporate the floating base dynamics equations of motion instead.

Xinjilefu et al. (2014) formulated the state estimation of the Atlas robot as a Quadratic Programming (QP) problem. The cost function was defined as the weighted sum of two quadratic terms: the modeling error and the measurement error, where the former is derived from the floating base dynamics equation of motion while the latter is derived from encoders, force/torque sensors, and IMU measurements. The optimization variable was composed of the generalized (i.e., joint and base link) velocities, the generalized forces, and the modeling error itself. Note that the base link pose was not part of the state and was estimated separately with an EKF. Tests on the Atlas robot have shown significant improvements in the behavior of the feedback controller with this estimation method.

A more unified optimization-based solution was proposed by Bloesch et al. (2018). In their approach, they eliminated the process model and made each measurement dependent on both the current and the previous state of the system. Intuitively, this is similar to an incremental smoothing method with a window of size two. The approach was able to integrate the dynamic equations of motion to estimate the linear and angular acceleration of the robot body in addition to what was sensed by the IMU, providing extra redundancy. If a process model were available, it could still be incorporated as a pseudo measurement, allowing the form of an EKF to be retained if required.

### 2.2. Multi-Sensor Filtering

Chilian et al. (2011) were among the first to discuss stereo, inertial, and kinematic fusion on a legged robot. They used a six-legged crawling robot measuring just 35 cm across, yet combining all the required sensing on board.

Similarly, Ahn et al. (2012) addressed the 3D pose estimation of the humanoid robot Roboray, using an EKF-based SLAM technique. Their motion estimation pipeline contains a visual-inertial-kinematic odometry module and a visual SLAM module. The kinematic and visual odometry are used to update the IMU measurements within an EKF filter. These constitute the input of the visual SLAM algorithm, which performed loop closures and decrease the drift.

Hornung et al. (2010) applied Monte Carlo localization (MCL), a Bayes filtering approach that recursively estimates the posterior, to estimate the 6 DoF pose of the Nao humanoid robot. By fusing the measurements of a 2D LIDAR with a motion model, they estimated the pose of the robot's torso, including while climbing a miniature staircase.

Ma et al. (2016) proposed an error-state Kalman filter fusing a tactical grade inertial measurement unit with stereo visual odometry to produce a pose estimate for navigation tasks, such as path planning. The robot's kinematic sensing was only used when visual odometry failed. Their approach was focused on pose estimation and was not used within the robot's closed-loop controller. Their extensive evaluation (over thousands of meters) achieved 1% error per distance traveled.

In contrast to the above-mentioned methods, we aim to estimate both the pose and the velocity of the robot with multi-sensor fusion and use this estimate online inside the control loop. This is motivated by the fact that, for highly dynamic motions, the drift rate of proprioceptive estimators is unacceptable and requires the integration of other exteroceptive signals.

The estimator used in this work is based on a loosely-coupled EKF, an approach that has been previously applied to Micro Aerial Vehicles (MAVs) (e.g., Lynen et al., 2013; Shen et al., 2014).

### 2.3. Multi-Sensor Smoothing

Smoothing methods are well-established in the MAV community for tightly coupled visual-inertial navigation, partly due to the relatively low complexity of these machines (e.g., fewer degrees of freedom). The main advantage of smoothing is the ability to jointly use all (or part) of the past history of measurements to reduce the uncertainty around the full robot's trajectory.

In recent years, promising works have been released that apply these techniques to legged machines. Hartley et al. (2018b) proposed the first attempt to fuse leg odometry and IMU in a factor graph on the Cassie bipedal robot. They extended the state with the feet contact locations and defined two new factors to incorporate forward kinematics and impose a zero velocity constraint on the contact points of a foot. These were then combined with the pre-integrated IMU factor from Forster et al. (2017a). Hartley et al. (2018a) extended this work to include additional pose measurements from the SVO Visual Odometry system (Forster et al., 2017b). Both works were demonstrated on Cassie in controlled environments for a short period of time (<5 min).

Wisth et al. (2019) proposed a tightly coupled visual-inertial-legged system based on the iSAM2 solver running on the ANYmal robot. The method extracts Kanade-Lucas features from the stereo camera on a RealSense D435 camera and optimizes them as the landmarks in the graph. Leg odometry was integrated as relative pose factors obtained from the internal state estimator running on the robot (Bloesch et al., 2018). The method was demonstrated in extensive outdoor experiments in urban and industrial scenarios where dynamic occludants and textureless areas were present in the scene.

All of the above works were based on the assumption of a stationary point of contact. This assumption is violated every time there are slippages or deformations of the leg and/or the ground. Contact detection methods can help to reject sporadic slippage or deformation events. However, when these occur regularly, they need to be modeled.

Wisth et al. (2020) proposed a factor graph method that models contact non-linearities a bias term of the linear velocity measurements from leg odometry. This can reduce the inconsistency between leg and visual odometry and provide a more robust pose and velocity estimate.

## 3. Problem Statement

We wish to track the pose and velocity of an articulated floating base robot with two or more legs and equipped with an onboard IMU, joint sensing of position and torque, cameras, and LIDARs. In this paper, we will focus on the Atlas and Valkyrie 28-DoF humanoids and on the HyQ and ANYmal 12-DoF quadrupeds. The robots of the same type share the same kinematic tree, with differences only in the link lengths and sensor locations.

### 3.1. Frames and Definitions

**Figure 2:**
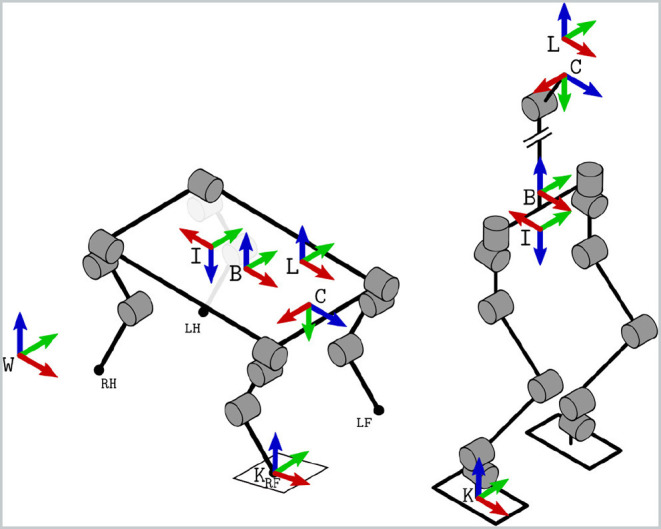
Reference frame conventions for typical quadruped and humanoid robots (with a simplified upper torso structure). The world frame *W* is fixed to earth, while the base frame *B*, the camera's optical frame *C*, and the IMU frame *I* are rigidly attached to the robot's chassis or head. When a foot touches the ground (e.g., the Right Front, RF), a contact frame *K* (perpendicular to the ground) is defined.

In Figure 2, we illustrate the reference frames relevant to our estimation problem. The inertial frame *W* and the base frame *B* are rigidly attached to the ground and the robot's floating base, respectively. The frames located at the sensor origins are also rigidly attached to the floating base, namely: the camera optical frame *C*, the IMU frame *I*, and the LIDAR frame *L*. The relative locations of these frames are known by design or can be retrieved with calibration procedures, such as the ones described in Furgale et al. (2013) and Reinke et al. (2019). One or more temporal contact frames *K* are created when a foot comes into contact with the ground.

#### 3.1.1. Notation

In the remainder of the paper, we adopt the following conventions: the robot position $\text{p}{=}_{W}{\text{p}}_{WB}\in {\mathbb{R}}^{3}$ and orientation **R** = **R**_*WB*_ ∈ SO(3) are from world to base and are expressed in world coordinates; the robot velocities $\text{v}{=}_{B}{\text{v}}_{WB},\omega {=}_{B}\omega_{WB}\in {\mathbb{R}}^{3}$ are from world to base, expressed in base coordinates; the IMU biases ${\;}_{I}{\text{b}}^{a}{,}_{I}{\text{b}}^{\omega }\in {\mathbb{R}}^{3}$ are expressed in IMU coordinates. A time-dependent vector quantity **a** computed at time *t*_*k*_ is shortened as **a**_*k*_ = **a**(*t*_*k*_).

### 3.2. State Definition

The robot state is defined as the vector combining position, orientation, linear velocity, and IMU biases. The angular velocity does not appear, as it is assumed to be directly measured by the IMU once properly bias compensated. The state at time *t*_*k*_ is:

(1) $$\begin{array}{l} x_{k}={\left[\begin{array}{ccccc} {\text{p}}_{k} & {\text{R}}_{k} & {\text{v}}_{k} & {\text{b}}_{k}^{a} & {\text{b}}_{k}^{\omega } \end{array}\right]}^{T} \end{array}$$

The orientation uncertainty is tracked by the exponential coordinates of the perturbation rotation vector, as described in Bry et al. (2012).

### 3.3. Requirements

To effectively track base and foot trajectories, the state estimate should have negligible drift, at least over the course of one planning cycle. Modern footstep planners typically replan every 1–5 s. Low-latency velocity estimates (including transduction and data transmission) are also fundamental for the feedback loop of a controller.

Low drift or drift-free state estimates are also required for navigation tasks (such as mapping and global path planning) as basic building blocks for many autonomous systems.

With these considerations in mind, we define the following requirements for a state estimator designed to run on legged robots in field operations:

- low pose drift in short range (e.g., 10 m);
- reliability in real semi-structured environments (i.e., does not diverge);
- signal smoothness for safe use in a control loop.

## 4. Method Description

Our approach adapts the core EKF filter described in Bry et al. (2012), with velocity corrections added by Fallon et al. (2014) for humanoid kinematics and then extended to quadruped kinematics in Camurri et al. (2017) (see section 4.2). Additional pose corrections from visual odometry and LIDAR registration are described in sections 4.4 and 4.5.

The goal of the EKF is to estimate the mean ***μ*** and covariance **Σ** of the Gaussian distribution over the state, ***x***_*k*_, given the previous state ***x***_*k*−1_, the current control input ***u***_*k*_, and the current measurement ***z***_*k*_. The state is first predicted using the non-linear discrete transition function ***f***(·) and then corrected by the observation function ***h***(·). Both functions are corrupted by zero-mean Gaussian process noise $w_{k}\sim N\left(0,{\text{Q}}_{k}\right)$ and measurement noise $\eta_{k}\sim N\left(0,{\text{P}}_{k}\right)$:

(2) $$\begin{array}{l} x_{k}=f\left(x_{k-1},u_{k},w_{k}\right) \end{array}$$

(3) $$\begin{array}{l} z_{k}=h\left(x_{k},\eta_{k}\right) \end{array}$$

The mean and covariance are propagated in the standard manner:

(4) $$\begin{array}{l} \mu_{k}^{-}=f\left(\mu_{k},u_{k},0\right) \end{array}$$

(5) $$\begin{array}{l} \Sigma_{k}^{-}={\text{A}}_{k-1}\Sigma_{k-1}{\text{A}}_{k-1}^{T}+{\text{W}}_{k-1}\text{Q}{\text{W}}_{k-1}^{T} \end{array}$$

where the minus superscript indicates that the quantity is evaluated before the measurement update takes place. For details on the derivation of the partial derivatives of the transition function, **A** and **W**, please refer to Bry et al. (2012).

The measurements are also integrated in a standard EKF manner. For instance, a velocity measurement ${\tilde{\text{v}}}_{k}\in {\mathbb{R}}^{3}$ with covariance matrix ${\text{P}}_{k}^{v}\in {\mathbb{R}}^{3\times 3}$ would be integrated as follows:

(6) $$\begin{array}{l} z_{k}={\tilde{\text{v}}}_{k} \end{array}$$

(7) $$\begin{array}{l} {\text{K}}_{k}=\Sigma_{k}^{-}{\text{H}}^{T}{\left(\text{H}\Sigma_{k}^{-}{\text{H}}^{T}+{\text{P}}_{k}^{v}\right)}^{-1} \end{array}$$

(8) $$\begin{array}{l} \mu_{k}=\mu_{k}^{-}+{\text{K}}_{k}\left(z_{k}-\text{H}\mu_{k}^{-}\right) \end{array}$$

(9) $$\begin{array}{l} \Sigma_{k}=\left(\text{I}-{\text{K}}_{k}\text{H}\right)\Sigma_{k}^{-} \end{array}$$

where ${\text{K}}_{k}\in {\mathbb{R}}^{15\times 3}$ is the Kalman gain and **H** ∈ ℝ^3 × 15^ is the Jacobian of the observation function, which in the specific case above acts as a selector matrix for the linear velocity substate.

### 4.1. Inertial Process Model

The acceleration (in the presence of gravity) and angular velocity are sensed by the IMU at high frequencies in the range 0.4–1 kHz. These are affected by bias and zero-mean Gaussian noise:

(10) $$\begin{array}{l} {}_{I}{\tilde{\omega }}_{WI}={\;}_{I}\omega_{WI}+{\text{b}}^{\omega }+\eta_{\omega } \end{array}$$

(11) $$\begin{array}{l} {}_{I}{\tilde{\text{a}}}_{WI}={}_{I}{\text{a}}_{WI}+{\text{b}}^{a}+\eta_{a} \end{array}$$

These quantities are transformed into the base frame and used as inputs to the process model:

(12) $$u=\left[\begin{array}{l} \omega \\ a \end{array}\right]=\left[\begin{array}{l} R_{\text{IB}}\left({\;}_{\text{I}}{\tilde{\omega }}_{\text{WI}}-b_{\omega }-\eta_{\omega }\right)\\ R_{\text{IB}}\left({\;}_{\text{I}}{\tilde{a}}_{\text{WI}}-b_{a}-\eta_{a}\right) \end{array}\right]$$

where **R**_*IB*_ is the rotational part of the rigid transform between the IMU and base frames. Note that we ignore the effects of angular acceleration and centripetal force (see Diebel, 2006) and assume that the IMU is close enough to the robot's base to make them negligible.

The process equations are:

(13) $$\begin{array}{l} \overset{∙}{\text{p}}=\text{R}\text{v} \end{array}$$

(14) $$\begin{array}{l} \overset{∙}{\text{R}}=\text{R}\omega^{\wedge } \end{array}$$

(15) $$\begin{array}{l} \overset{∙}{\text{v}}=-\omega^{\wedge }\text{v}+{\text{R}}^{T}\text{g}+\text{a} \end{array}$$

(16) $$\begin{array}{l} {\overset{∙}{\text{b}}}_{a}=\eta_{b}^{a} \end{array}$$

(17) $$\begin{array}{l} {\overset{∙}{\text{b}}}_{\omega }=\eta_{b}^{\omega } \end{array}$$

where $\eta_{b}^{\omega },\eta_{b}^{a}$ are bias random walk noises.

Given Equations (13)–(17), we can predict the mean of the state ***x***_*k*_ by simple integration over the period Δ*t* = *t*_*k*_−*t*_*k*−1_:

(18) $$\begin{array}{l} u_{k}=\left[\begin{array}{c} \omega_{k}\\ {\text{a}}_{k} \end{array}\right]=\left[\begin{array}{c} {\text{R}}_{IB}\left({\tilde{\omega }}_{k}-{\text{b}}_{k-1}^{\omega }\right)\\ {\text{R}}_{IB}\left({\tilde{\text{a}}}_{k}-{\text{b}}_{k-1}^{a}\right) \end{array}\right] \end{array}$$

(19) $$\begin{array}{l} \mu_{k}^{-}=f\left(\mu_{k-1},u_{k},0\right)=\left[\begin{array}{c} {\text{p}}_{k-1}\\ {\text{v}}_{k-1}\\ 0\\ {\text{b}}_{k-1}^{a}\\ {\text{b}}_{k-1}^{\omega } \end{array}\right]+\\ \;\left[\begin{array}{c} {\text{v}}_{k-1}\Delta t\\ \left(-{\tilde{\omega }}_{k}^{\wedge }{\text{v}}_{k-1}+{\left({\text{R}}_{k-1}\right)}^{T}\text{g}+{\text{a}}_{k}\right)\Delta t\\ {\text{R}}_{k-1}\exp\left(\omega_{k}^{\wedge }\Delta t\right)\\ 0\\ 0 \end{array}\right] \end{array}$$

Note that the attitude is integrated separately using the exponential map between the Lie group of rotations and its Lie algebra at the identity (see Forster et al., 2017a).

The prior covariance $\Sigma_{k}^{-}$ is also computed by Euler integration of the partial derivatives of the process equation, as detailed in Bry et al. (2015).

Having propagated the filter, measurements from other sensors can be used to correct the state vector. In the following sections, we derive measurements and their covariance matrix from leg, visual, and LIDAR odometry.

### 4.2. Leg Odometry

Leg odometry estimates the incremental motion of the floating base of a legged robot from the forward kinematics of the legs in stable contact with the ground. This measurement can be formulated as either a relative pose or a velocity measurement. In our system, we formulate linear velocity measurements.

In the following sections, we derive this measurement specifically for humanoids and quadrupeds.

#### 4.2.1. Humanoids

We adopt the contact classification and velocity measurement strategies from Fallon et al. (2014).

##### 4.2.1.1. Contact Classification

Humanoid robots are typically equipped with force/torque sensors at the feet, from which the contact state can be inferred by thresholding the measured normal force. Torsional friction is assumed to be high enough for there to be no foot rotation.

We use a Schmitt trigger to classify contact forces sensed by the robot's three-axis foot force-torque sensors and to detect how likely a foot is to be in contact. For simplicity, only one foot is detected as in contact during a double support phase and a simple state machine is used to decide which foot is more reliable.

We also classify other events in the gait cycle, such as striking contact (as a 20–30 N positive and increasing discontinuity lasting more than 5 ms) and breaking contact (negative force discontinuity below a threshold). Because these events create unrealistic measurements, the EKF integrates them with higher measurement covariance.

Finally, we found that, in some cases, it is necessary to use the state of the controller to decide which contact points are in stable contact. For example, when climbing stairs, the toe of the trailing foot pushes the robot upward but is not in stationary contact (a “toe off” event). In that case, we use information from the controller to assign the leading foot to be the primary fixed foot.

##### 4.2.1.2. Measurements

Once the primary fixed foot is established, a velocity measurement is created by differentiation of the base position across the interval Δ*t* = *t*_*k*_−*t*_*k*−1_. The foot contact locations at times *t*_*k*−1_ and *t*_*k*_ are defined as the composition of the base position in world coordinates and the foot position in base coordinates:

(20) $$\begin{array}{l} {}_{W}{\text{p}}_{WK}\left(t_{k-1}\right)={\text{p}}_{k-1}+{\text{R}}_{k-1}\text{fk}\left({\text{q}}_{k-1}\right) \end{array}$$

(21) $$\begin{array}{l} {}_{W}{\text{p}}_{WK}\left(t_{k}\right)={\text{p}}_{k}+{\text{R}}_{k}\text{fk}\left({\text{q}}_{k}\right) \end{array}$$

(22) $$\begin{array}{l} {}_{W}{\text{p}}_{WK}\left(t_{k}\right)={}_{W}{\text{p}}_{WK}\left(t_{k-1}\right) \end{array}$$

where fk(·) is the forward kinematic function that returns the foot location in base coordinates and **q** are the joint positions.

Since the contact location in world coordinates does not change over the interval (see Equation 22), the difference in position **p**_*k*_−**p**_*k*−1_ can be inferred from the forward kinematics only, by subtracting and rearranging Equations (20)–(21). Finally, the discrete differentiation is then simply computed by dividing **p**_*k*_−**p**_*k*−1_ by the time interval (Equation 23).

(23) $$\begin{array}{l} {\tilde{\text{v}}}_{k}=\frac{{\text{p}}_{k}-{\text{p}}_{k-1}}{\Delta t}+\eta^{v}=\\ \;=\frac{{\text{R}}_{k-1}\text{fk}\left({\tilde{\text{q}}}_{k-1}\right)-{\text{R}}_{k}\text{fk}\left({\tilde{\text{q}}}_{k}\right)}{\Delta t}+\eta^{v} \end{array}$$

(24) $$\begin{array}{l} z_{k}={\tilde{\text{v}}}_{k} \end{array}$$

where $\tilde{\text{q}}$ are the measured joint positions and the covariance matrix ${\text{P}}_{k}^{v}={\text{P}}^{v}$ is defined from fixed values (empirically found) that are increased when special events (striking contact, breaking contact) occur.

#### 4.2.2. Quadrupeds

Quadruped robots are typically equipped with high-precision joint encoders from which low-noise joint velocity measurements can be derived. However, achieving accurate contact estimation is a major challenge since field-ready quadrupeds are not typically equipped with direct contact sensors, as they easily break during operation.

##### 4.2.2.1. Contact Classification

Quadruped robot feet are usually approximated to be point-like and then assumed to exert only pure forces onto the ground. These forces can be estimated for each individual leg ℓ∈{LF, RF, LH, RH} using the base acceleration $\overset{∙}{\omega },\overset{∙}{\text{v}}$ and torques ***τ***:

(25) $$\begin{array}{l} {\text{f}}^{\ell }=-{\left(\text{J}{\left(\text{q}\right)}^{T}\right)}^{†}\left(\tau -{\text{h}}_{q}-F^{T}\left[\begin{array}{c} \overset{∙}{\omega }\\ \overset{∙}{\text{v}} \end{array}\right]\right) \end{array}$$

where J(·) is the foot Jacobian, **h**_*q*_ are the Coriolis effects, and *F* is the matrix of spatial forces required at the floating base to support unit accelerations about each joint variable (see Featherstone, 2008).

Let $f_{k}^{\ell }\in \mathbb{R}$ be the vertical component of **f**^ℓ^∈ℝ^3^ at time *t*_*k*_. Thus, we model the probability for a particular foot being in *firm, static, and stable* contact with the following Sigmoid function:

(26) $$\begin{array}{l} P_{k}^{\ell }\left(s_{k}^{\ell }=1|{\text{f}}_{k}^{\ell }\right)=\frac{1}{1+\exp\left(-\beta_{1}f_{k}^{\ell }-\beta_{0}\right)} \end{array}$$

where $s_{k}^{\ell }\in B$ is a binary value that indicates contact/no-contact for foot ℓ at time *t*_*k*_. We learn the Sigmoid parameters β_0_ and β_1_ using a logistic classifier, as described in Camurri et al. (2017).

For each leg, we determine a (binary) contact state $s_{k}^{\ell }=1$ if $P_{k}^{\ell }>0.5$, and $s_{k}^{\ell }=0$ otherwise.

##### 4.2.2.2. Measurements

Having determined the set of legs in contact, for a given leg ℓ the robot's linear velocity can be computed as follows:

(27) $$\begin{array}{l} {}_{B}{\text{v}}_{WB}=-{}_{B}{\text{v}}_{BK}-{}_{B}\omega_{WB}\times {}_{B}{\text{p}}_{BK} \end{array}$$

From the sensed joint positions and velocities $\tilde{\text{q}},\tilde{\overset{∙}{\text{q}}}$ and their additive noises $\eta^{q},\eta^{\overset{∙}{q}}$, we can rewrite Equation (27) as a linear velocity measurement of the robot's base, computed using the leg ℓ:

(28) $$\begin{array}{l} {\tilde{\text{v}}}_{\ell }=-\text{J}\left(\tilde{\text{q}}-\eta^{q}\right)\cdot \left(\tilde{\overset{∙}{\text{q}}}-\eta^{\overset{∙}{q}}\right)-\omega \times \text{fk}\left(\tilde{\text{q}}-\eta^{q}\right) \end{array}$$

where fk(·) and J(·) are the forward kinematics function and its Jacobian, respectively.

As in Bloesch et al. (2013), we collect all the effects of noise into one additive term **η**^*v*^:

(29) $$\begin{array}{l} {\tilde{\text{v}}}_{k}^{\ell }=-J\left({\tilde{\text{q}}}_{k}\right){\tilde{\overset{∙}{\text{q}}}}_{k}-\omega \times \text{fk}\left({\tilde{\text{q}}}_{k}\right)+\eta^{v} \end{array}$$

Since multiple legs can be in contact simultaneously, we define the velocity measurement as a weighted average using the set of legs in contact, where weights are determined using the contact probabilities $P_{k}^{\ell }$ from Equation (26):

(30) $$\begin{array}{l} {\tilde{\text{v}}}_{k}=\frac{\sum P_{k}^{\ell }{\tilde{\text{v}}}_{k}^{\ell }}{\sum P_{k}^{\ell }}+\eta^{v}\;\forall \ell |s^{\ell }\neq 0 \end{array}$$

(31) $$\begin{array}{l} z={\tilde{\text{v}}}_{k} \end{array}$$

The adaptive covariance ${\text{P}}_{k}^{v}$ is associated with the velocity measurement and accounts for harsh impact forces (up to 600 N for a 90-kg robot trotting). These forces can severely undermine the estimation performance, because compression of the legs or the ground causes incorrect kinematic measurements, which translate into velocity and position errors.

The covariance is computed as the combination of a fixed term (from the encoder noise datasheet), the inter-leg velocity covariance **D**_*k*_, and a term that is proportional to force discontinuities (that are caused by impacts). For convenience, let *c*_*k*_ be the total number of detected contact legs at time *t*_*k*_. The inter-leg covariance is defined as the covariance matrix of the velocity contributions from the contact legs:

(32) $$\begin{array}{l} {\text{D}}_{k}=\frac{1}{c_{k}}\sum ({\tilde{\text{v}}}_{k}-{\tilde{\text{v}}}_{k}^{\ell }){\left({\tilde{\text{v}}}_{k}-{\tilde{\text{v}}}_{k}^{\ell }\right)}^{T}\\ \;≃\left[\begin{array}{ccc} \sigma_{x}^{2} & 0 & 0\\ 0 & \sigma_{y}^{2} & 0\\ 0 & 0 & \sigma_{z}^{2} \end{array}\right]=\Lambda \left(\sigma_{x}^{2},\sigma_{y}^{2},\sigma_{z}^{2}\right) \end{array}$$

The force discontinuity is defined as the mean absolute difference of the normal force for each leg:

(33) $$\begin{array}{l} \Delta f=\frac{1}{c_{k}}\underset{\forall \ell }{\sum }|f_{k}^{\ell }-f_{k-1}^{\ell }| \end{array}$$

From Equations (32)–(33), the final covariance for the velocity measurement is:

(34) $$\begin{array}{l} {\text{P}}_{k}^{v}={\text{P}}_{0}^{v}+{\left[\frac{1}{2}\left(\Lambda \left(\sigma_{x},\sigma_{y},\sigma_{z}\right)+{\text{I}}_{3}\frac{\Delta f}{\alpha }\right)\right]}^{2} \end{array}$$

where α is a constant normalization factor, empirically determined.

### 4.3. Zero Velocity Bias Estimation

The yaw drift due to bias evolution can be significant over long periods of time. Yaw error is also the dominant source of drift in any state estimator or SLAM system. For this reason, in Ma et al. (2016), zero velocity updates were used to measure rotation rate bias estimates.

In our system, we continually check for periods where the robot is stationary using the joint velocities and GRF.

When the robot is stationary for at least 400 ms, the gyro bias is updated to the average angular velocity recorded during the stationary period:

(35) $$\begin{array}{l} {\tilde{\text{b}}}_{k}^{\omega }=\frac{1}{t_{k}-t_{i}}\overset{k}{\underset{j=i}{\sum }}{\tilde{\omega }}_{j} \end{array}$$

(36) $$\begin{array}{l} z_{k}={\tilde{\text{b}}}_{k}^{\omega } \end{array}$$

where *t*_*k*_−*t*_*i*_> 400 ms.

Since the bias is generally a very small quantity (i.e., ***ω***≫**b**^ω^), the covariance associated with the measurement can typically be set to very small values without affecting the control system of the robot.

### 4.4. Visual Odometry

Visual Odometry estimates the pose of the robot by tracking features on camera images. The VO estimate frequency is typically in the range of 10–30 Hz, which corresponds to the camera frame rate.

When used in combination with LIDAR odometry, the benefits of VO are two-fold. First, it makes the overall estimated trajectory smoother when compared with a inertial-kinematic-LIDAR-only system, as it reduces the drift rate between two LIDAR updates. Second, the reduced drift rate also helps the LIDAR registration itself, as the sparsity of the LIDAR scans requires the accumulation of scans over time before performing the registration. Therefore, a lower drift rate during the accumulation produces higher-quality point clouds to be registered.

Our visual odometry pipeline is based on the FOVIS algorithm by Huang et al. (2011). The measurements are loosely integrated into the filter as relative pose measurements between frames. This would allow the use of other VO algorithms, such as ORB-SLAM (Mur-Artal et al., 2015), SVO (Forster et al., 2017b), or VINS-Mono (Qin et al., 2018), to name a few. FOVIS was chosen because of its computational efficiency.

The only input to FOVIS is a sequence of stereo image pairs. It tracks FAST features in a key frame approach to estimate incremental camera motion. Given two keyframes at times *t*_*i*_, *t*_*j*_, we denote the estimated relative motion of the camera between these two times as ${\;}_{C_{i}}{\tilde{\text{T}}}_{C_{i}C_{j}}={\tilde{\text{T}}}_{C_{ij}}$. Using the known camera-to-base frame transformation, _*B*_**T**_*BC*_, this can be expressed in the corresponding estimate of the motion of the base frame as:

(37) $$\begin{array}{l} {\tilde{\text{T}}}_{B_{ij}}{=}_{B}{\text{T}}_{BC}\;{\tilde{\text{T}}}_{C_{ij}}{\left({}_{B}{\text{T}}_{BC}\right)}^{-1} \end{array}$$

We integrate the VO estimate for a time window *t*_*j*_−*t*_*i*_, which is typically 2–3 s. When used in combination with the LIDAR module, we then form a position measurement in the world frame as follows:

(38) $$\begin{array}{l} {}_{W}{\tilde{\text{T}}}_{WB}\left(t_{j}\right)={}_{W}{\text{T}}_{WB}\left(i\right){\tilde{\text{T}}}_{B_{ij}} \end{array}$$

(39) $$\begin{array}{l} {\tilde{\text{p}}}_{j}=\text{trans}({}_{W}{\tilde{\text{T}}}_{WB}(t_{j})) \end{array}$$

(40) $$\begin{array}{l} z_{j}={\tilde{\text{p}}}_{j} \end{array}$$

where the pose of the robot at time *t*_*i*_ is taken from the filter's history of states. Note that we choose to use only the translational part of Equation (38) for the EKF filter update, as yaw corrections from the LIDAR are more accurate and sufficiently frequent.

Without the LIDAR module, the VO update can also include rotational components (typically only yaw, since roll and pitch are observable from the IMU).

Note that the update could be delayed in time (i.e., *t*_*j*_ < *t*_*k*_), so the filter will re-apply the chain of measurements from time *t*_*j*_ to *t*_*k*_, as explained in section 5.1.

The covariance matrix for the measurement was manually set to fixed values. However, when the FOVIS algorithm reports failure, the measurement is discarded. The algorithm reports failure in three cases: (1) when the number of inlier features being tracked is below a threshold (10 in our case); (2) when the solution of the optimization is degenerate; (3) when the reprojection error is higher than a threshold (1.5 pixels in our case).

**Figure 3:**
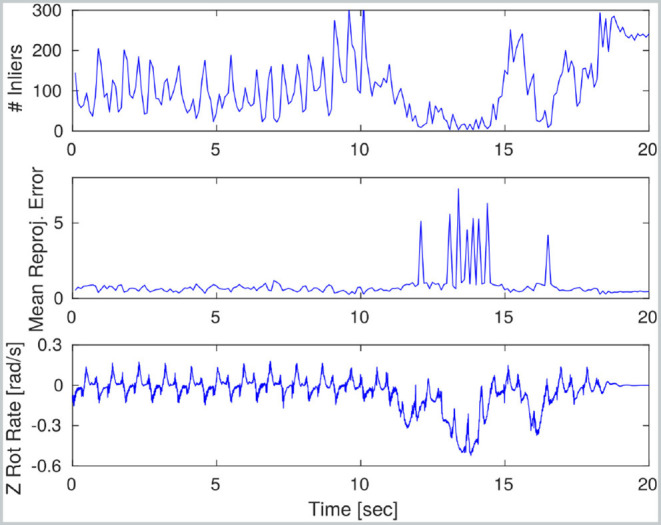
Visual odometry performance during a trotting sequence on HyQ: the robot first trots forward at 0.3 m/s and then turns in place sharply over a 5 s period. During the initial trotting phase, VO performance is satisfactory. However, image blur causes the number of inliers to fall and mean re-projection error to spike. During this part of the experiment, no VO measurements are incorporated into the main motion estimate.

An example of failure is provided by Figure 3, where at time 12 s the number of inliers drops below the threshold (top plot) and the reprojection error increases significantly (medium plot) due to an abrupt robot rotation that caused motion blur (bottom plot).

### 4.5. LIDAR Odometry

Our LIDAR odometry is based on the Auto-tuned Iterative Closest Point (AICP) algorithm by Nobili et al. (2017b), which improves the ICP implementation from Pomerleau et al. (2013) by making it more robust against significant changes in overlap between the clouds to be registered.

The rotating Hokuyo LIDAR sensor inside the Multisense SL (mounted on Atlas, Valkyrie, and HyQ), as well as the Velodyne VLP-16 (mounted on ANYmal), produces very sparse point clouds that cannot be used directly for scan to scan registration.

Therefore, we accumulate consecutive measurements from the sensor as a *reference* point cloud. The filter's state is used as the source of robot poses during the accumulation. We assume that the pose drift during the accumulation is small enough not to create significantly distorted reference point clouds. In this context, the VO module is important, as it keeps the drift bounded during the accumulation period.

Once a sufficiently dense reference cloud is obtained, a sequence of *reading* point clouds are accumulated and registered against the reference for motion estimation. The result of the registration constitutes an additional relative pose measurement for the EKF.

#### 4.5.1. Reference Update

Using the first accumulated point cloud as the reference and registering the forthcoming clouds as reading is effective only in confined scenes. When the robot travels far away from its initial location, this method is intractable due to the decreasing overlap between the source and the reading clouds, eventually resulting in ICP failure.

To guarantee sufficient overlap between the reference and the reading clouds, we update the reference clouds whenever the overlap drops below a safety threshold. In long-range missions, such as the one described in section 7.5, we conveniently forced a reference update after the robot had traveled 5 m from its initial location. This way, the drift is effectively bounded while having sufficient overlap for data association.

#### 4.5.2. Pre-filtering

According to Segal et al. (2009), point-to-plane registration has proven to have superior performance to point-to-point. Therefore, we extract planar macro-features (e.g., walls, doors, ceilings) and implicitly discard all other entities (including dynamic obstacles). We also apply a voxel filter with a leaf size of 8 cm to uniformly downsample the clouds. This step is necessary to equalize the contribution from all of the points during the optimization process, as point clouds are denser in the proximity of the sensor.

**Figure 4:**
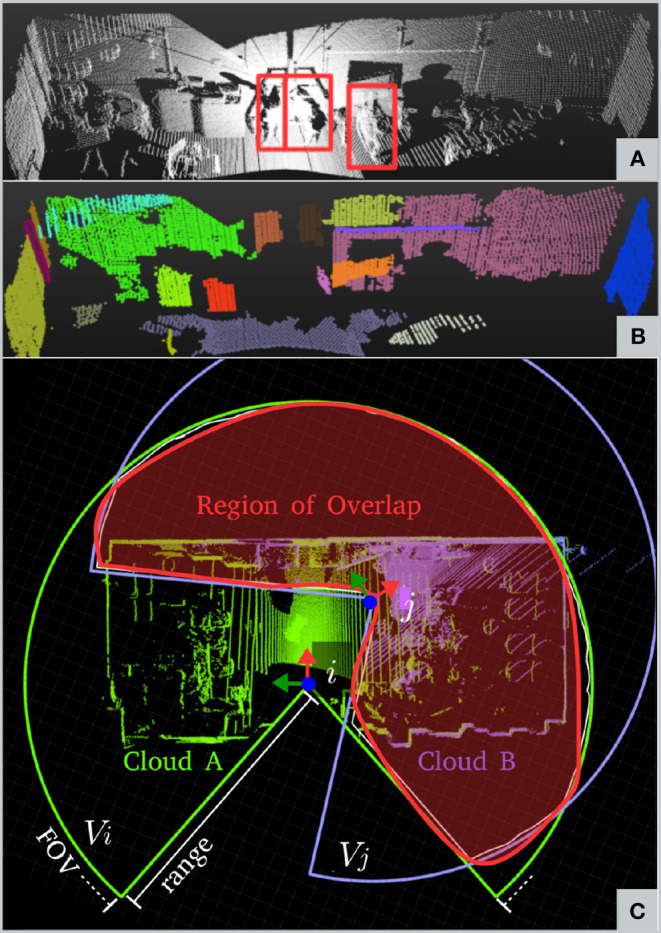
Pre-filtering and Outlier filtering. **(A)** Raw point cloud from Valkyrie's dataset; people are outlined in red. **(B)** After pre-filtering; people and small irrelevant features have been filtered out. **(C)** Region of overlap as the result of the analysis of the volumes of the two point clouds.

For planar surface extraction, we adopt a region growing strategy: a patch that is larger than a specific area (e.g., 30 × 30 cm) is accepted for further process. An example of output pre-filtering is shown in Figures 4A,B.

#### 4.5.3. Auto-Tuned ICP

Most ICP solutions assume a constant overlap between reference and reading clouds. However, when partial occlusion occurs (e.g., during passage through a narrow door), this assumption is violated and the massive concentration of points on the occludant (e.g., the walls beside the door) can cause incorrect correspondences.

In contrast, we continuously estimate the amount of overlap between the point clouds and automatically tune the ICP inlier ratio for robust registration. The overlap parameter is proportional to the true positive correspondences (i.e., the higher Ω is, the larger the number of true positive matches and vice versa). In the following subsection, we briefly describe how Ω is computed. More details can be found in Nobili et al. (2017b).

#### 4.5.4. Overlap Filter

The overlap parameter Ω is computed in a point-wise fashion (Figure 4C). Let _*W*_*A*,_*W*_*B* be the reference and reading point clouds acquired at times *t*_*i*_, *t*_*j*_ whose points have been expressed in world coordinates by using a prior from the EKF. Each cloud is confined into the volumes *V*_*i*_, *V*_*j*_ by the sensor Field of View (FoV). The intersection of the two volumes defines an overlap region (red in the figure). If *S*_*i*_ and *S*_*j*_ are the points of *A*_*i*_ and *B*_*j*_ belonging to the overlap region, we can define the overlap parameter Ω as:

(41) $$\begin{array}{l} \Omega =\frac{\left|S_{i}\right|}{\left|A\right|}\cdot \frac{\left|S_{j}\right|}{\left|B\right|} \end{array}$$

where |·| indicates the number of points in the cloud.

We use the overlap parameter from (41) to dynamically set the inlier ratio of the ICP algorithm. If 0.2 < Ω <0.7, we set the inlier ratio to Ω. If Ω is below 0.2, the inlier ratio is set to 0.2, as this is the minimum required for ICP registration. Finally, if Ω exceeds 0.7, the inlier ratio is bounded to 0.7 to avoid overestimation.

We follow three heuristics to determine whether an alignment is successful. First, the mean residual point-wise error should be smaller than the threshold α:

(42) $$\begin{array}{l} \text{MSE}=\frac{1}{n}\overset{n}{\underset{i=1}{\sum }}r_{i}<\alpha \end{array}$$

where *r*_1_, …, *r*_*n*_ are the residual distances between the accepted matching points in the input clouds. Second, the median of the residual distribution, *Q*(50), should be smaller than the threshold α:

(43) $$\begin{array}{l} Q\left(50\right)<\alpha \end{array}$$

Third, the quantile corresponding to the overlap measure should also be smaller than α:

(44) $$\begin{array}{l} Q\left(\Omega \right)<\alpha \end{array}$$

The first two conditions are commonly used metrics of robustness, while the third automatically adapts to the degree of point cloud overlap. The parameter α was set to 0.01 m during our experiments.

#### 4.5.5. Measurements

Once the two clouds *A, B* have been successfully registered, the relative pose estimate ${\tilde{\text{T}}}_{B_{ij}}$ of the robot's base between times *t*_*i*_ and *t*_*j*_ is available, similarly to Equation (37). Thus, the measurement is incorporated in the same way but including rotation:

(45) $$\begin{array}{l} {}_{W}{\tilde{\text{T}}}_{WB}\left(t_{j}\right)={}_{W}{\text{T}}_{WB}\left(i\right){\tilde{\text{T}}}_{B_{ij}} \end{array}$$

(46) $$\begin{array}{l} {\tilde{\text{p}}}_{j}=\text{trans}({}_{W}{\tilde{\text{T}}}_{WB}(t_{j})) \end{array}$$

(47) $$\begin{array}{l} {\tilde{\text{R}}}_{j}=∠({}_{W}{\tilde{\text{T}}}_{WB}(t_{j})) \end{array}$$

(48) $$\begin{array}{l} z_{j}=\left[\begin{array}{cc} {\tilde{\text{p}}}_{j} & {\tilde{\text{R}}}_{j} \end{array}\right] \end{array}$$

where again the absolute pose of the robot at time *t*_*i*_ is taken from the filter and the covariance matrix is set to fixed values. Note that the time index is *j* as, typically, the measure is delayed (i.e., *t*_*j*_ < *t*_*k*_).

## 5. Implementation

**Figure 5:**
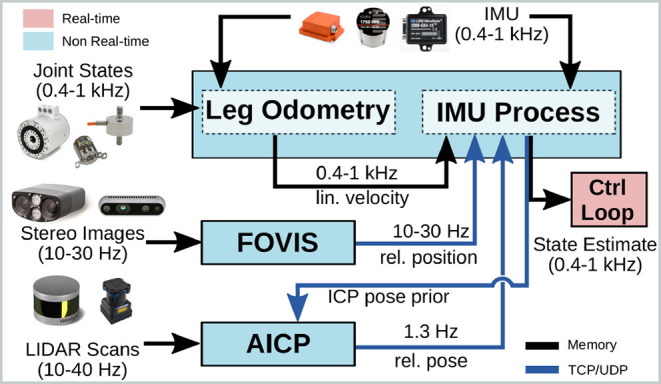
Block diagram of our system: the IMU process model and leg odometry are run on the control computer in a real-time process (in pink), while the other modules run on separate computers in the user space (light blue). These modules output filter measurements as ROS messages, which are exchanged with the real-time domain through native shared memory mechanisms.

A block diagram of our system is presented in Figure 5. Even though the Pronto modules can all be run on a single machine, it is common practice in legged robot design to distribute the computation across two separate computers: a Control PC connected to actuators and proprioceptive sensors running a real-time operating system and a Vision PC for exteroceptive sensor processing. This design architecture has been adopted for all the robots evaluated in this paper. Its main advantage is that the more critical operations are unaffected by potential delays, failure, or overloads caused by the resource-intensive processing of data from camera and LIDAR.

Therefore, the IMU prediction and leg odometry updates are performed within the same UNIX process running on the Control PC. After every IMU process step, the estimator immediately shares the filter state with the control system via a real-time interface based on shared memory. The same estimate is also available on the network for other modules to use (e.g., as a prior for ICP).

The FOVIS and AICP processes are run on the Vision PC. Both modules are decoupled from the core estimator, which receives the updates as timestamped messages via a TCP or UDP channel (e.g., ROS messages). This allows Pronto to perform the core IMU/leg odometry, which is more critical, and to incorporate measurements if and when they are available.

On all platforms, the computation is carried out on consumer-grade processors (e.g., equivalent to Intel i7 for a laptop), with no need for GPU processing.

### 5.1. Measurement History

The implementation of the filter maintains a history of measurements (with their covariance), filter prior/posterior states, and filter covariances, covering a time window of typically 10 s. This allows the incorporation of asynchronous corrections from VO and LIDAR, which have significant latency.

**Figure 6:**
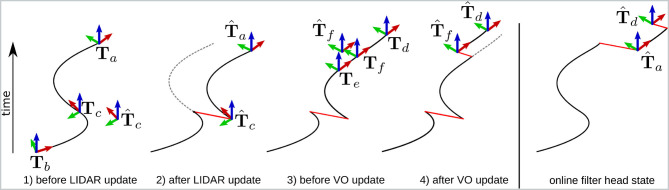
Example illustrating how VO and LIDAR measurements can be incorporated into the filter despite having much higher latency than the IMU process model. In black is the best estimate of the trajectory at that instant, in red are updates introduced by incorporated measurements, and dashed gray lines are parts of the trajectory that are recomputed. For clarity, the magnitude of the corrections is exaggerated. Elapsed time is indicated in the upward direction.

In Figure 6, we explain the concept with a toy example. In black is the best estimate of the current state and history at that moment in time. In red are discontinuities caused by EKF updates (exaggerated for clarity). In dashed gray are portions of the filter history that are overwritten due to a received measurement.

**Event 1:** At time *t*_*a*_, the head of the filter points to **T**_*a*_. This state is the result of predictions and measurement integrations available up to time *t*_*a*_. At this same time, the filter receives a delayed LIDAR measurement with timestamp *t*_*c*_ (with *t*_*c*_ < *t*_*a*_). In particular, the measurement involves the relative pose between **T**_*b*_ and **T**_*c*_ (with *t*_*b*_ < *t*_*c*_). The history consists of a window of measurements, filter states, and filter covariances, ordered by timestamp. Since the window is longer than the time interval *t*_*a*_ − *t*_*b*_, the filter head can be moved back to **T**_*c*_, which corresponds to the state recorded at time *t*_*c*_.

**Event 2:** The LIDAR measurement is incorporated as an EKF correction, resulting in the posterior estimate ${\hat{\text{T}}}_{c}$. At this point, all the measurements in the history with timestamps after *t*_*c*_ are re-applied to the filter as if they had been received after the LIDAR measurement. As a result, the filter head at time *t*_*a*_ becomes ${\hat{\text{T}}}_{a}$. The past trajectory (dashed gray line) is therefore overwritten. The new current state ${\hat{\text{T}}}_{a}$ is the same as it would have been if the LIDAR measurement had been received at time *t*_*c*_ instead of *t*_*a*_.

**Event 3:** Over the next period of time, the filter continues to propagate the head of the estimator using the IMU process model and leg odometry. At time *t*_*d*_, a new visual odometry measurement is created that measures the relative transformation of the body frame between time *t*_*e*_ and time *t*_*f*_. This measurement is typically received with a 150–300 ms delay.

**Event 4:** We wish to use this information to correct the pose of the robot toward ${\hat{\text{T}}}_{f}$, as described in section 4.4. The key step is that this correction to the filter is carried out using the re-filtered trajectory (mentioned in Event 2). After the correction is applied, the head of the filter becomes ${\hat{\text{T}}}_{d}$, and the estimator continues as normal.

The final sub-figure (on the right) shows the state of the head of the filter over the course of the example. This is the running estimate that would have been available to the controller online.

Note that the proposed framework qualifies as an odometry system, as no loop closures are performed. Therefore, typical corrections from the exteroceptive modules are in the order of a few centimeters (*cf*. **Figure 9C** top right). These discontinuities are small enough to be dealt with by the position controller acting on the robot base with appropriate gains. Bigger discontinuities, such as the ones from a SLAM system, are typically addressed by using two different reference frames for control and for global path planning [e.g., Meeussen (2010)].

### 5.2. Software Structure

The framework presented in this paper is available to the research community at three open-source repositories:

- pronto^1^: library implementations of the EKF inertial process model and the Leg Odometry modules described in sections 4.1 and 4.2, respectively
- fovis_ros^2^: ROS wrapper for the FOVIS algorithm (previously open-source but not ROS-compatible)
- aicp_mapping^3^: implementation of the AICP algorithm described in section 4.5.

The first repository is independent from the others and contains all the code necessary to implement a proprioceptive state estimator on a legged robot. To deploy the algorithm on a legged robot of choice, either with or without ROS, the implementation of the forward kinematics API and the creation of a dedicated executable is required. A complete example of a deployment on the ANYmal robot is also provided.

## 6. Experimental Platforms

In the following sections, we describe the relevant characteristics of the experimental platforms used: the Atlas and Valkyrie humanoid robots and the HyQ and ANYmal dynamic quadruped robots. A summary of the main sensors mounted on the robots is provided in Table 1.

**Table 1:** Sensor specifications divided by robot.

**Sensor**	**Model**	**Hz**	**Specs**
**Atlas**			
IMU	KVH 1750	333	*Init Bias:* 0.5 °/h | 0.5 mg
			*Bias Stab:* 0.05 °/h | 0.05 mg
Stereo Camera	Multisense SL	10	*Res:* 1024 × 1024 px
			*FoV:* 80 × 80°
			*Imager:* CMV4000 4MP
LIDAR	Hokuyo UTM-30LX-EW	40	*FoV (full rot.):* 220 × 180°
Encoder	N/A	333	*Res:* < 0.0045°
Torque	N/A	333	*Res:* N/A
**Valkyrie**			
IMU	3DM-GX4-25	500	*Init Bias:* 0.05 °/s | 2 mg
			*Bias Stab:* 10 °/h | 0.04 mg
Stereo Camera	Multisense SL	10	*FoV (full rot.):* 180 × 120°
Encoder	N/A	500	*Res:* 0.0043°
F/T	ATI Omega85	500	*Res:* 0.07–0.1 N
			0.02–0.03 N m
**HyQ**			
IMU	KVH 1775	1,000	*Init Bias:* 0.5 °/h | 0.5 mg
			*Bias Stab:* 0.05 °/h | 0.05 mg
Stereo Camera	Multisense SL	10	See above
Encoder	AEDA3300-BE1	1,000	*Res:* < 0.0045°
Force	Burster 8417	1,000	*Res:* < 25 N
Torque	N/A	1,000	*Res:* N/A
**ANYmal**			
IMU	Xsens MTi-100	400	*Init Bias:* 0.2 °/s | 5 mg
			*Bias Stab:* 10 °/h | 15 mg
Stereo Camera	RealSense D435	30	*Res:* 848 × 480 px
			*FoV:* 91.2 × 65.5°
			*Imager:* IR global shutter
Encoder	ANYdrive	400	*Res:* < 0.025°
Torque	ANYdrive	400	*Res:* < 0.1 N m

### 6.1. Atlas

Atlas (version 5, Figure 1A) is a 195 cm high, 95 kg, 28-DoF hydraulic robot manufactured by Boston Dynamics for the DARPA Robotics Challenge. Each leg has six joints (three hip, one knee, and two ankle joints), the position of which are estimated from the measured travel of their hydraulic actuators using a Linear Variable Differential Transformer (LVDT). Since the accuracy of these devices is limited, the joint velocities are very noisy and are therefore not used directly for leg odometry (*cf*. section 4.2.1). Other measurement non-linearities, such as backlash have been addressed the same way as Koolen et al. (2016).

Located at the pelvis is a tactical KVH 1750 IMU equipped with a Fiber Optic Gyro (FOG) for accurate angular velocity measurements.

The main source of exteroceptive signals is the Carnegie Robotics Multisense SL, a tri-modal ruggedized sensor that includes a rotating Hokuyo UTM-30LX-EW, a high-quality rolling shutter RGB stereo camera with a 7 cm baseline, and an FPGA implementation of the stereo Semi-Global Matching algorithm by Hirschmüller (2008) to provide dense 3D point clouds at nominal camera frequency. All these signals are synchronized in hardware through the FPGA. The laser produces 40 line scans per second with a 30 m maximum range while spinning about the forward-facing axis. Every few seconds, it spins half a revolution and a full 3D point cloud is accumulated with a Field of View (FoV) of 220 × 180°.

### 6.2. Valkyrie

Valkyrie (Figure 1B) is a 1.87 m tall, 129 kg, 44-DoF (28-DoF without hands) electrically actuated robot developed by NASA for the DARPA Robotics Challenge and space operations Radford et al. (2015). As for Atlas, each leg has 6 DoF, with 3-DoF hips, 1-DoF knee, and 2-DoF ankles. The hip and knee motors are rotary actuators whose rotation is measured by magnetic encoders and whose torque is quantified by measuring the spring deflection. The ankle joints are linear, with encoders located along the axis of rotation for joint position measurement, and load cells located on the shaft for torque measurement, respectively.

Even though the robot is equipped with several cameras for visual servoing, the main exteroceptive sensor considered in this paper is again the Multisense SL. The FoV of the LIDAR is reduced to 180 × 120° by a plastic cover over the head.

### 6.3. HyQ

HyQ (Figure 1C) is a torque-controlled Hydraulic Quadruped robot developed by Semini et al. (2011) at the Istituto Italiano di Tecnologia (IIT). The system is 1 m long and weighs ~85 kg. Its 12 revolute joints have a rotational range of 120° and a maximum torque of 160 N m. The 1 kHz sensors are read by a control computer (using a real-time operating system). All other sensors are connected to a perception computer and are passively synchronized with the real-time sensors as described in Olson (2010).

As for Atlas and Valkyrie, the robot's main exteroceptive sensor is the Carnegie Robotics Multisense SL. The stereo camera was configured to capture 1,024 × 1,024 images at 10 Hz. **Figure 10** shows an example of a left camera image and a depth image taken during an experiment, indicating the challenging scenarios we target.

### 6.4. ANYmal

ANYmal (version B, Figure 1D) is a 12-DoF electrically actuated quadruped robot initially designed by Hutter et al. (2016) at ETH Zurich and now manufactured by ANYbotics. It is 80 cm long and weighs 33 kg. Its series elastic actuators can deliver up to 40 N m of torque and provide accurate measurements of the joint position, velocity (internally computed by differentiation), and torque (by spring deflection measurement).

The robot is equipped with an XSens MTi-100 industrial-grade IMU, a RealSense D435 camera at the front (for visual stereo odometry and local mapping), and a Velodyne VLP-16 LIDAR on the top (for localization and global mapping).

## 7. Experimental Results

We carried out a series of experiments with the Atlas, Valkyrie, HyQ, and ANYmal robots over the course of 4 years. We present summary results, which have a combined time of 2 h and 13 min and 1.37 km of distance traveled, respectively. A summary of the experimental results divided by dataset and robot is available in Table 2.

**Table 2:** Summary of the experiments.

**Exp. N**	**Robot**	**RPE**	**VO**	**AICP**	**OL**	**CL**	**T**	**DT**	**A**	**GT**
		**[m]**					**[s]**	**[m]**	**[m2]**	
1	Atlas	≤ 0.03*	–	✓	✓	✓	1,236	16	154	–
2	Valkyrie	0.016	–	✓	✓	✓	341	12	78	✓
3	Valkyrie	0.016	–	✓	✓	✓	50	2.5	78	✓
4	HyQ	0.027	✓	✓	✓	✓	1,740	400	7.5	✓
5	HyQ	≤ 0.03**	✓	✓	✓	✓	1,740	400	9	–
6	HyQ	0.033	✓	✓	✓	✓	2,640	300	100	✓
7	ANYmal	0.34	✓	✓	–	–	1,996	240	1,381	✓
		0.83	–	–	–	–				

*Exp. N, Experiment number; RPE, Relative Pose Error (translational part, evaluated over 10 m distance); ATE, Absolute Translation Error; VO, Visual Odometry; OL, Online; CL, Control Loop; T, Time; DT, Distance Traveled; A, Area; GT, Ground Truth*.

* *By evaluation of the ground truth point cloud*.

** *By evaluation of the accuracy in returning to the initial position*.

### 7.1. Evaluation Protocol

We aim to evaluate the estimation performance both quantitatively and qualitatively, with a focus on real-world scenarios and online/real-time execution.

#### 7.1.1. Ground Truth

The experimental results presented in this section have been collected over the span of several years in a variety of different conditions and platforms. For this reason, it was not always possible to generate the ground truth poses from the same source (e.g., motion capture). The last column of Table 2 indicates the experiments where ground truth was available.

For all indoor experiments on HyQ and Valkyrie (lines 2–4, Table 2), we used a Vicon motion capture system to achieve millimeter-accurate ground truth poses at 100 Hz.

For the HyQ outdoor experiments (line 6 in the table), we exploited situations where the robot was completely stationary to accumulate six full sweeps of LIDAR scans from different locations to reconstruct the scene in post-processing via ICP registration. Since the LIDAR was perfectly stationary, the accumulation was performed for at least two full turns (65k points per scan), and the overlap was more than 70 %, we ascribe the accuracy of the reconstruction to one of the sensors, which is 3 cm for the experimental area evaluated. Then, we generated a ground truth trajectory by aligning the point cloud data from the onboard LIDAR with the prior map in post-processing. Note that this trajectory is different than the one obtained during online estimation, as there was no prior map involved in this process.

The experiment with ANYmal (line 7, Table 2) have been paired with ground truth from a Leica TS16 laser tracking system, which tracked the robot's position with millimeter accuracy using a reflective prism on the robot. The data from the laser tracker was then spatio-temporally aligned with the IMU to get ground truth poses via an offline batch optimization, as described in Burri et al. (2016).

Finally, when ground truth was not available, we designed the experiments such that the estimation performance could be measured by analyzing the robot's accuracy in returning to its initial position after several forward/backward motions.

#### 7.1.2. Pose Estimation Performance

Since our proposed algorithm is an odometry system (i.e., no loop closures are preformed), we base our quantitative analysis on the mean translational component of the Relative Pose Error (RPE), defined by Sturm et al. (2012), over a distance of 10 m. The performance for each experiment is indicated in Table 2.

#### 7.1.3. Control Loop Performance

We evaluated the stability of the algorithm in real conditions by running the estimator in real-time on Valkyrie (to feed the footstep planner). On Atlas and HyQ, we also closed the control loop with the estimator. The control loop test implicitly evaluates the quality of the velocity estimates, which are directly used by the locomotion controllers. For ANYmal, the execution was tested offline but at nominal speed and on a consumer-grade laptop with comparable performance to the hardware mounted on the robot. In this case, the suitability for the control loop was assessed by looking at the signal smoothness.

### 7.2. Atlas Experiments

The Atlas dataset (Table 2, Experiment 1) was collected during a run by the MIT team at the DARPA Robotics Challenge Finals (Pomona, CA 2015). It consists of 20 min 36 s of continuous operation in a semi-structured environment measuring 14 × 11 m, with walls on the right side of the robot and an open-space populated by a crowd on the left. The robot walks through the test scenario along a 16 m path while passing over uneven terrain and manipulating objects (Figure 1A). Accurate maps of the environment were obtained in post-processing.

During the whole competition, Pronto (without AICP) was used to close the control loop. Its low-drift estimation performance (evaluated to be ~1.67 % traveled distance in preliminary indoor tests) allowed it to successfully traverse uneven and rough terrain, although pauses for re-localization were necessary.

**Figure 7:**
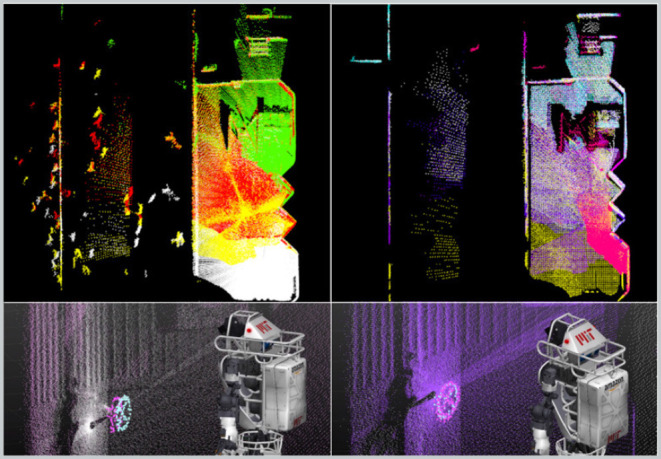
AICP performance on the DRC Finals dataset with Atlas. (Top) A top view of the alignment of 206 point clouds during the run—left: raw clouds with people, right: filtered clouds. Bottom left: state estimation without applying correction, valve perceived in different locations by successive clouds. Bottom right: with successful localization, consistent estimate of the affordance.

Later on, further offline tests were carried out after the integration of the AICP module. This time, the system performance was qualitatively evaluated from careful observation of the map after the run (Figure 7), where the estimated trajectory is close to error-free (~3 cm error for the full run). People have been filtered out and do not contribute to the alignment (top right). The algorithm is stable and robust enough to compute successful alignments during the entire run (with more than 14 m displacement and overlap decreasing to just 10 %), satisfying requirement 2. Under the same conditions, standard ICP algorithms fail after 400 s, as they are not accounting for dynamically changing point cloud overlap across the run.

### 7.3. Valkyrie Experiments

The state estimation framework was tested online on two different tasks: repeated walking on flat ground (Table 2, Experiment 2) and stair climbing (Table 2, Experiment 3).

#### 7.3.1. Repeated Walk to a Target

**Figure 8:**
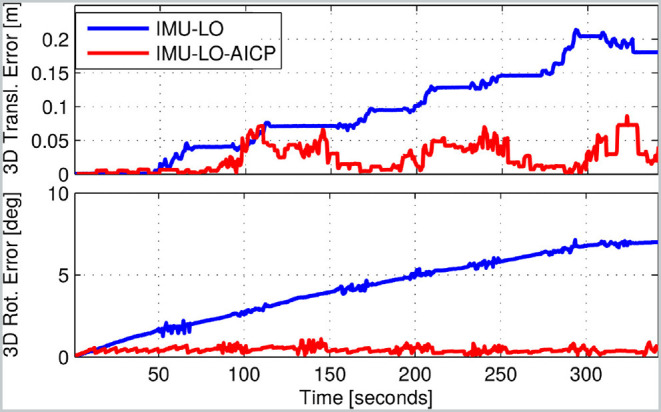
Translational and rotational error for Experiment 2 (Valkyrie). The blue line shows the kinematic-inertial typical estimation drift, while in red is the estimate with the AICP corrections.

Valkyrie walked repeatedly forward toward a fixed target identified at the beginning of the run before reversing direction. Over the course of the experiment, the error in translation never exceeded 7.5 cm and was 1.6 cm on average, whereas the estimator without LIDAR had an unbounded drift (Figure 8), mostly dominated by yaw bias (see bottom plot). This satisfies the requirements about expected localization accuracy. Thanks to this localization performance, the robot could reach the goal target and maintain a precise pose estimate during the entire run. In contrast, using the proprioceptive state estimator, the robot failed to reach the target due to odometry drift.

#### 7.3.2. Stair Ascend

Valkyrie was placed at 1 m from a staircase. The task was to walk toward it and climb up the steps. Planning was performed only once, at the start. Over the course of this 50 s experiment, the median errors in translation and rotation were comparable to those in Experiment 2. This level of accuracy allowed the robot to safely perform the task without needing to re-plan. In contrast, during the DRC, robots typically took a few steps at a time to climb stairs or traverse uneven terrain, pausing periodically to manually re-localize and re-plan. In this context, our system was demonstrated to enable greater autonomy in task execution.

### 7.4. HyQ Experiments

**Figure 9:**
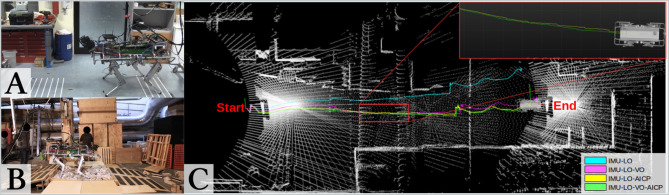
**(A)** Indoor repeatability tests. **(B)** Outdoor exploration tests in challenging scenarios. **(C)** Comparison between estimated trajectories of HyQ from Experiment 6d: IMU and Leg Odometry (cyan); IMU, Leg Odometry, VO (magenta); IMU, Leg Odometry (LO), AICP (yellow); IMU, LO, VO, AICP (green). Note that the IMU-LO-VO-AICP trajectory is smoother than the combination without VO (inset).

On HyQ, we performed experiments in two different scenarios. First, for Experiment 4, a repetitive trotting motion was carried out in a laboratory environment with a Vicon motion capture system for ground truth. Second, for Experiments 5 and 6, extensive testing was carried out in a poorly lit industrial area with a featureless concrete floor, as well as test ramps and rock beds (Figure 9B). The environment, the different locomotion gaits (trotting and crawling), and the uneven terrains presented a large number of challenges to our algorithms and demonstrated the importance of using redundant and heterogeneous sensing. The robot's peak velocity when trotting was about 0.5 m/s, which is approximately half of typical human walking speed.

**Figure 10:**
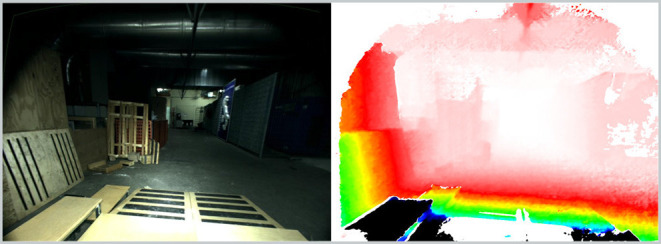
Example of left camera image and depth image produced by HyQ's stereo camera. This reflects the difficult lighting conditions and challenging structure of the test arena. The scene is illuminated with the sensor's on-board lights.

#### 7.4.1. Indoor Repeated Trot to a Target

The robot was commanded to continuously trot forward and backward to reach a fixed target (a particular line in Figure 9A). Robot position and velocity estimates were used by the controller to stabilize the robot motion while tracking the desired position, as described in Barasuol et al. (2013).

Periodically, the operator updated the target so as to command the robot to trot a further 10 cm forward. The experiment continued for a total duration of 29 min. At the end of the run, the robot had covered a total distance of about 400 m and trotted forward and backward 174 times.

In Table 2, we show that the drift is below 3 cm when combining IMU, Legged, Visual, and LIDAR odometry. By comparison, without any exteroceptive signals, the drift was more than three times higher. When testing the addition of VO or LO independently, we noticed that incorporating VO reduces the drift rate relative to the baseline system, while adding AICP achieves drift-free localization, since the AICP re-localizes against the same fixed map (the room).

To test the performance with uneven terrain and where the reference point cloud has to be updated due to longer paths, a second series of experiments was carried out in a larger environment.

#### 7.4.2. Outdoor Repeated Trot to a Target

An equivalent experiment was performed within a section of a 20 m × 5 m industrial area surrounded by pallets, walls, and air treatment machines. The robot repeated a forward-backward motion covering a 6 m × 1.5 m area toward a target placed at 5 m distance from its starting position (Figure 1C). The robot traveled about 400 m at a 0.5 m/s trotting gait, reaching the target 40 times without any user input at run time.

The results presented in this section show that the fully integrated state estimation system, leveraging IMU, leg odometry, VO, and AICP data, produced a very low-drift estimate of the robot state. However, no LIDAR reference cloud updates were triggered, as the robot did not travel far from its initial location.

In the case of larger explorations, every reference update generates an accumulated error. The magnitude of this error depends on the residual error from the alignment of the new reference to the previous. In the case of HyQ, a reference update happens once every 10–13 m distance covered, depending on occlusions. In the following section, we present statistics from experiments where multiple LIDAR reference cloud updates were made.

#### 7.4.3. Outdoor Industrial Area Exploration

The robot explored the same industrial area described in the previous section. To test the system in different conditions, in some experiments, we have added rough terrain and ramps (Figure 9B), with both crawling and trotting gaits at up to 0.5 m/s. Turning in place (as seen in Figure 3) represented an extra challenge for the state estimation system. Lighting conditions varied dramatically during data recording, from bright light to strong shadows and from day to night-time. In some experiments, on-board lighting was used. The dataset is summarized in Table 3 and consists of five runs, for a total duration of 44 min and 300 m traveled.

**Table 3:** Detailed summary of the dataset used for Experiment 6, including log duration, size of arena, type of motion (F/B = forward/backward trajectory), laser spin rate, and terrain features.

**N**	**Gait**	**Duration (s)**	**Area (m^**2**^)**	**Laser (RPM)**	**Ramp**
6a	Crawl	869	20 × 5, F/B	5	✓
6b	Crawl	675	20 × 5, F	5	✓
6c	Trot	313	20 × 5, F/B	15	X
6d	Trot	330	20 × 5, F/B	10	X
6e	Trot	469	7 × 5, F/B	10	✓

No motion capture system was available in this space: to quantitatively evaluate the state estimation performance on the dataset, we built a prior map made up of a collection of four carefully aligned point clouds, and we estimated drift relative to that.

##### 7.4.3.1. Crawling gait

In the previous section, we showed (while trotting) that integrating VO reduces the pose drift rate between the lower-frequency AICP corrections. Here, we focus on the importance of using VO in addition to AICP.

**Figure 11:**
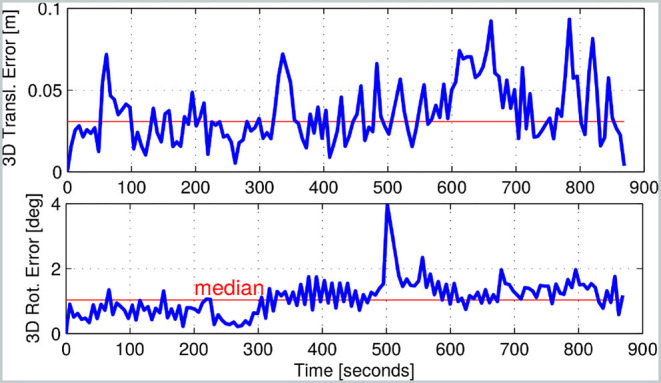
Estimated error of the state estimator used in Experiment 6a. The experiment involved the robot crawling for a total of 40 m.

Figure 11 shows the estimated error over the course of Experiment 6a, recorded in the arena of Figure 9. The robot started from pose A, reached B, and returned to A. The robot crawled for 40 m and paused to make three sharp turns. The experiment was at night and used the on-board LED lights.

During this run, the reference point cloud was updated four times. After 860 s, the state estimation performance had not significantly degraded, despite no specific global loop closure being computed.

##### 7.4.3.2. Trotting gait

As mentioned previously, trotting is a more dynamic gait with a higher proprioceptive drift rate, which means that the VO could better contribute when combined with AICP. Empirically, this can be seen in the inset plot in Figure 9. In this case, the algorithm with VO produces a smoother trajectory (in green) than without (in yellow). This is important because the robot's base controller uses these estimates to close position and velocity control loops. Discontinuities in the velocity estimate could lead to undesired destabilizing foot forces and controller reactions.

In brief, for Experiments 6c–6e, the integration of AICP allowed state estimation with an average 3D median translation error of ~4.9 cm. The integration of VO further reduced the median translation error to 3.2 cm (Figure 11). The RPE over 10 m is in line with the indoor experiments.

### 7.5. ANYmal Experiments

**Figure 12:**
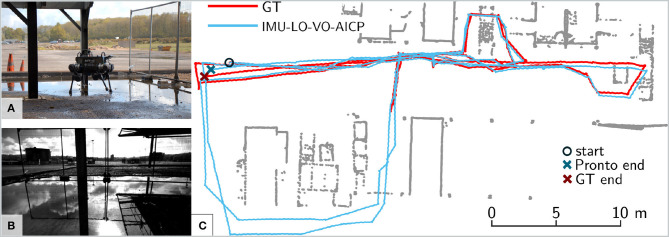
**(A)** Experiment 7 with the ANYmal robot at the Fire Service College. **(B)** Onboard camera feed during the experiment. Note the challenging conditions for visual odometry due to reflections in the water. **(C)** Estimated trajectory from Pronto with FOVIS and AICP active (blue) against ground truth (red). The ground truth was not available in the bottom area. Start and end locations for both algorithms are highlighted with a circle and a cross, respectively.

The ANYmal dataset was collected at the Fire Service College, a 32.5 × 42.5 m industrial oil rig facility used for firefighter training (Figure 12). The ground truth was collected with a laser tracking system, a Leica TS16, which tracked the robot's position with mm accuracy using a reflective prism on the robot.

The robot started from an open area and was commanded to trot at 0.3 m/s inside the facility, between metal containers and stairs, performing three loops before returning to the initial position, for a total of 240 m distance covered in 33 min. The dataset includes several extra challenges in addition to the ones in the previous section: (1) the area covered is much wider, so we had to trigger forced AICP reference updates on a regular basis (i.e., once every 0.5 m traveled); (2) the scene includes open areas where the robot looks at the horizon, where a very limited number of stereo features are available; (3) the scene contains reflections due to water puddles, which confuse the visual feature tracking.

The different level of performance compared to previous tests is due to several factors related to the scenario used. In contrast with the previous experiments, the open space and the size of the area covered force triggering of frequent reference updates (more than 40 updates vs. four updates in Experiment 6 on HyQ). As no loop closures are performed, in this situation, the LIDAR cannot completely eliminate the drift accumulated when new reference updates are triggered. In addition, the Velodyne scans are much sparser due to the wider scenario (only a few LIDAR rings are projected onto the ground), making it hard to constrain the robot position on the *z*-axis. We have partially compensated for this problem by augmenting the LIDAR data with a filtered output of a downward-facing RealSense D435.

Despite these challenges, the system is able to effectively fuse all the sensors modalities, achieving an RPE of 34 cm over 10 m, which corresponds to 3.4 % error. The contribution of the LIDAR localization is particularly evident on the *z*-axis, where it significantly reduces the characteristic vertical drift caused by leg/ground compression while trotting. This allowed the RPE to be reduced by 60 % from the baseline algorithm with IMU and Leg Odometry only. After traveling 240 m, the pose estimate is <30 cm away from the ground truth (*cf*. the estimate on the *xy*-plane in Figure 12C).

## 8. Discussion

In the previous section, we demonstrated the ability of our system to overcome a variety of perception challenges, including low light conditions, motion blur, reflections, dynamic motions, and rough terrain. We also showed its versatility by demonstrating its support of a variety of sensor modalities and four different legged robots.

The simple but effective integration of delayed signals into the time history described in section 5.1 allowed us to integrate two different odometry sources (Visual and LIDAR) despite their significant delay and different frequencies.

A limitation of the current approach is the lack of measurement update triaging in case of disagreement between different exteroceptive sources. Currently, when an exteroceptive module does not report failure, confidence in the measurement is only encoded by a fixed covariance matrix. A possible alternative approach is to implement a mechanism that maps the error metrics specific to a module (e.g., VO reprojection error, ICP registration error) into a dynamically changing covariance matrix.

Alternatively, transitioning from loosely to tightly coupled approaches would allow joint optimization over all of the measurements, making the estimation more robust against outlier updates. This is ongoing work.

## 9. Conclusion

We have presented a state estimation framework to perform sensor fusion of inertial, kinematic, visual sensing, and LIDAR on legged robots, built upon a modular Extended Kalman Filter.

In particular, we showed how our approach supports dynamic maneuvers and operation in sensor-impoverished situations. The reliability of our approach was demonstrated with dynamic gaits and speeds of up to 0.5 m/s. A particular technical achievement has been reliably closing the loop with this state estimator in dynamic gaits.

During experiments lasting over 2 h, our system was demonstrated to be robust and continuously accurate, with an RPE of <35 cm over 10 m traveled for the most challenging scenario and 2–3 cm in smaller areas.

Our current filter marginalizes out previous state variables. In future work, we will explore using windowed smoothing to incorporate measurements relative to previous filter states. We are also interested in extending the state with dynamic quantities, such as CoM and linear/angular momenta similarly to Xinjilefu et al. (2014).

## Data Availability Statement

The datasets generated for this study are available on request to the corresponding author.

## Author Contributions

MC wrote the main article, implemented the quadruped leg odometry and the overall filter architecture, and ran the experiments on HyQ. MR performed the LIDAR localization experiments on the ANYmal robot. SN implemented the LIDAR localization algorithm and performed the localization experiments on Atlas, Valkyrie, and HyQ. MF implemented the bipedal leg odometry, revised, and approved the manuscript.

## Conflict of Interest

The authors declare that the research was conducted in the absence of any commercial or financial relationships that could be construed as a potential conflict of interest.

## Footnotes

^1^https://github.com/ori-drs/pronto

^2^https://github.com/ori-drs/fovis_ros

^3^https://github.com/ori-drs/aicp_mapping
